# Development of an Effective Treatment Algorithm for the Stubbed Great Toe

**DOI:** 10.7759/cureus.17246

**Published:** 2021-08-17

**Authors:** Taylor A Harris, Jacqueline Krumrey, Jennifer Sharp

**Affiliations:** 1 Orthopedics, Good Samaritan Regional Medical Center, Corvallis, USA; 2 Orthopedics and Traumatology, Good Samaritan Regional Medical Center, Corvallis, USA

**Keywords:** open, phalanx, toe, pediatric, fracture, osteomyelitis

## Abstract

While toe fractures are the most common fractures of the foot in the pediatric population, the majority of these injuries do not require aggressive treatment. However, the mechanism of stubbing the great toe with bleeding at the base of the nail, a laceration proximal to the nail fold, or signs of a displaced fracture through the physis of the distal phalanx signal a likely open fracture involving the distal phalanx of the great toe. Unrecognized open fractures can lead to mistreatment and consequently osteomyelitis or growth disturbances. We report a case of a patient who required open reduction and Kirschner wire (K-wire) stabilization of a Salter-Harris I fracture involving the distal phalanx of the great toe after delayed recognition of the initial injury and subsequent failure to obtain closed reduction with a digital block in the clinic setting. We present this case to demonstrate the importance of vigilance in the evaluation of stubbed great toe injuries and propose an algorithm to guide the management of these injuries.

## Introduction

Injuries involving a stubbed great toe in the pediatric population are often managed non-operatively with overall successful outcomes and few complications [1]. However, without an appropriate level of suspicion, significant injuries can go unrecognized and mistreated. Physical examination findings such as bleeding at the base of the toenail, a laceration proximal to the nail fold, and a displaced physeal fracture of the distal phalanx indicate a high probability of an open fracture [2,3]. While appropriate initial management with antibiotics and treatment of the fracture typically progresses to an uneventful recovery, failure to recognize the significance of the open injury can lead to long-term consequences such as osteomyelitis, growth arrest and resulting deformity, and loss of range of motion [4,5].

The importance of acute recognition and appropriate management of the open fracture is well established; however, there is scarce literature detailing consensus of management and stabilization of open Salter-Harris fractures of the distal phalanx of the great toe. Due to the intimate relationship of the epiphyseal growth plate and the germinal matrix, there is a risk for the interposition of the germinal matrix within the fracture, which could block reduction. These types of injuries have been closely compared to Seymour fractures of the terminal phalanx of the finger in which the treatment algorithm involves antibiotic administration, open reduction of the fracture, irrigation and debridement of the fracture site, nailbed repair, and possible Kirschner wire (K-wire) fixation across the distal interphalangeal joint (DIPJ) for maintenance of reduction [6]. In available case reports, open fractures have demonstrated the ability to be successfully managed with closed reduction and nailbed repair following a digital block in the emergency department or clinic [4,5]. We report the case of a patient who required open reduction and K-wire stabilization of a Salter-Harris I fracture involving the distal phalanx of the great toe after delayed recognition of the initial injury and subsequent failure to obtain closed reduction with a digital block in the clinic setting. We present this case to demonstrate the importance of vigilance in consultation and evaluation of stubbed great toe injuries and propose an algorithm to guide the management of these injuries.

## Case presentation

**Figure 1:**
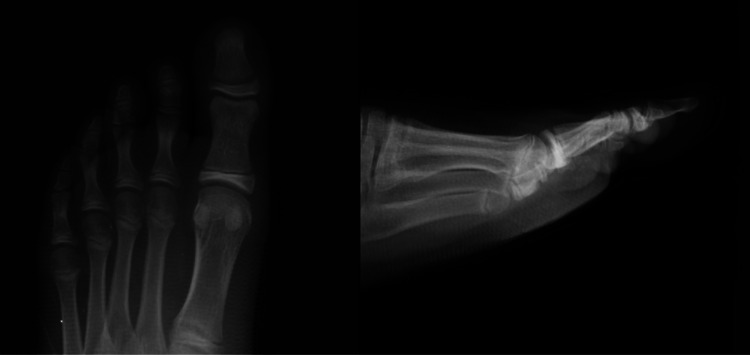
AP (left) and lateral (right) radiographs of the left foot in a 13-year-old male after stubbing his great toe, which demonstrates a Salter-Harris I fracture of the distal phalanx with dorsal displacement, plantar angulation of about 10°, and widening of the dorsal aspect of the physis. AP, anteroposterior.

A healthy 13-year-old male presented to the orthopedic clinic five days after injuring his left great toe while running on the stairs. Initially, after the injury, bleeding was noted at the base of the nail which, lasted several hours, and he felt something hard “like bone” protruding from the wound. He was seen at an urgent care clinic the day following his injury, where radiographs of the left great toe were obtained, demonstrating a displaced Salter-Harris type I fracture of the distal phalanx of the great toe with dorsal displacement and angulation resulting in widening of the dorsal aspect of the physis (Figure 1).

**Figure 2:**
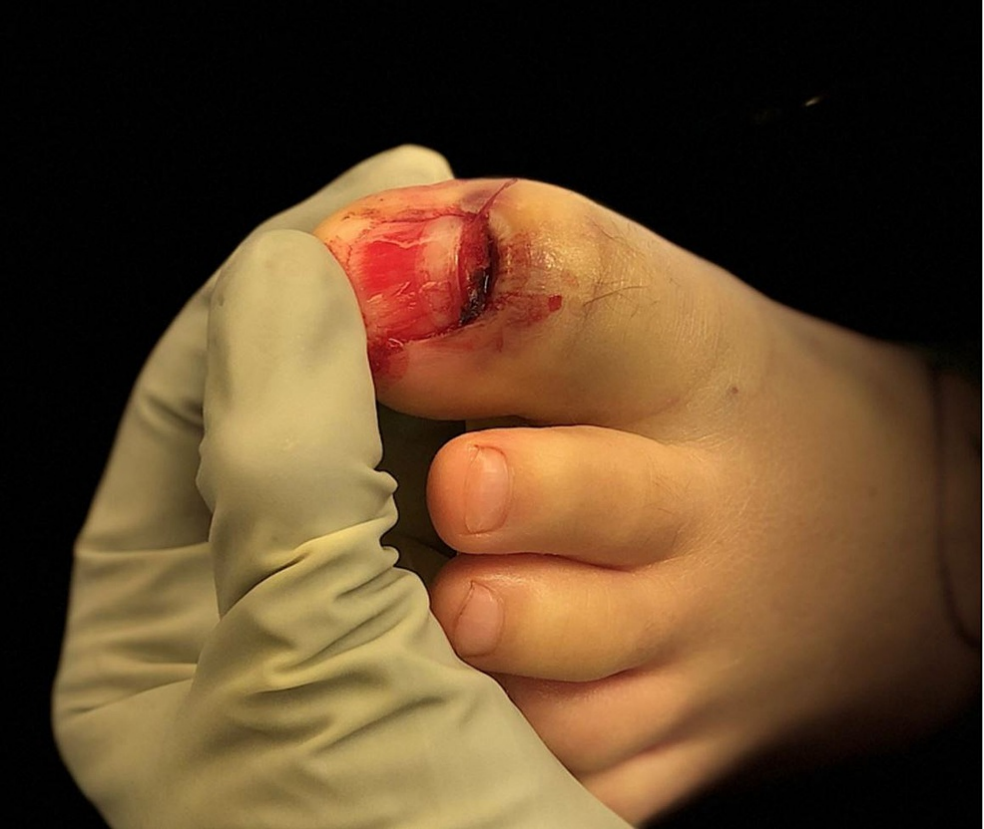
Intra-operative photograph of the left great toe demonstrating a dorsal laceration and avulsion injury of the proximal nail bed with exposed fracture and germinal matrix.

The wound was soaked in warm water and chlorhexidine and dressed in bacitracin and a sterile bandage, but a reduction attempt was not performed. After consultation with an on-call orthopedic provider regarding follow-up recommendations and confirming his tetanus was up-to-date, he was discharged home with a post-op shoe, a prescription for oral cephalexin, and instructions to follow-up with the acute orthopedic clinic within one week. Upon presentation in the orthopedic clinic, he reported tolerable pain and no change in sensation. Physical examination demonstrated a decreased range of motion at the great toe interphalangeal joint and a dorsal laceration over the interphalangeal joint of his great toe with exposed germinal matrix (Figure 2).

**Figure 3:**
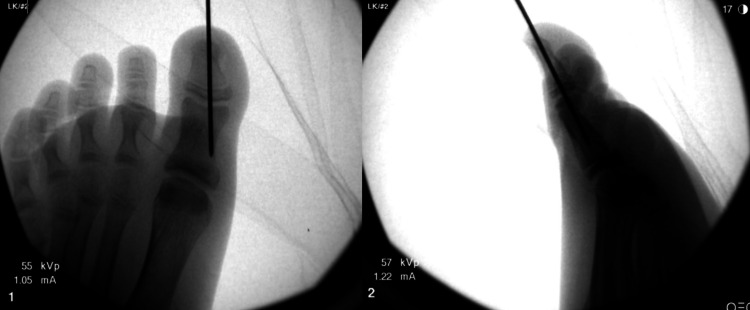
Intra-operative AP (left) and lateral (right) fluoroscopic radiographs of the left great toe demonstrating reduction and stabilization of a Salter-Harris I fracture of the distal phalanx with a Kirschner-wire. AP, anteroposterior.

Following an unsuccessful attempt at reduction under digital block in the orthopedic clinic, the decision was made to proceed with urgent operative intervention for management of the patient’s open Salter-Harris I fracture with an interposed germinal matrix. Intraoperatively, the patient underwent nail removal where it was noted the germinal matrix was completely interposed within the physeal separation. To retract the interposed tissue, two 5-mm back cut incisions were made on the medial and lateral borders of the eponychial fold. Then, the proximal aspect of the eponychium was elevated dorsally and the matrix was exposed. The fracture was cleared of interposed tissue, irrigated with sterile saline, then reduced and stabilized with a 0.054 Kirschner-wire (K-wire) (Figure 3). The nail was then replaced following the reduction and stabilization of the fracture.

**Figure 4:**
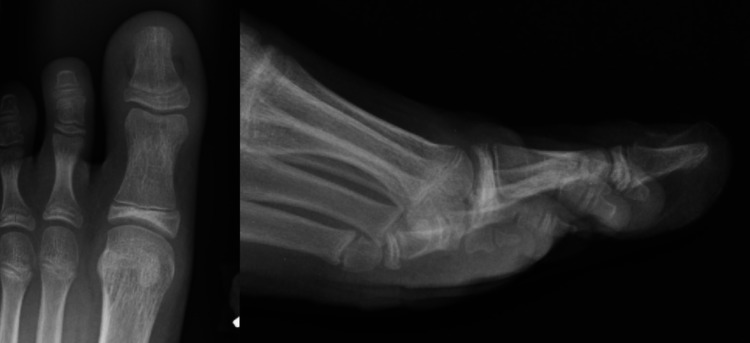
AP (left) and lateral (right) radiographs of the left great toe six weeks post-operatively demonstrating interval removal of the Kirschner-wire and maintenance of reduction with healing of the Salter-Harris I fracture of the distal phalanx. AP, anteroposterior.

The patient was allowed to be weight-bearing as tolerated after surgery in a post-op shoe and instructed to complete the full course of cephalexin previously prescribed for a total course of 10 days. He followed up one week post-operatively, and his wounds appeared to be healing well without signs of infection. He was instructed to follow up at six weeks post-operatively for K-wire removal. He followed up three weeks post-operatively for evaluation after accidental removal of the pin at home. Radiographs at that time demonstrated appropriate healing of the fracture, and physical examination did not demonstrate concern for infection. He remained in a post-op shoe for protection until six weeks post-operatively. Final radiographs at that time demonstrated good healing with anatomic alignment, and he was able to transition into a regular shoe and activities without complication (Figure 4). The patient was instructed to follow up at three months post-operatively but the appointment was canceled because he did not have any concerns and he has not required subsequent re-evaluation.

## Discussion

Stubbing injuries of the great toe place the distal phalanx into forced hyperflexion and are often associated with physeal injuries of the distal phalanx. The consequence of a missed open physeal fracture of the great toe from a stubbed toe injury has been well described. Pinckney et al. [2] originally described this injury as a unique entity after observing the development of cellulitis and osteomyelitis in children who sustained stubbed great toe injuries with underlying fractures through the physis of the distal phalanx. They determined that the relationship of the bone and nail is responsible for an increased likelihood for open fractures to occur. At the dorsum of the distal phalanx, the skin attaches directly to the periosteum, and a displaced fracture of the physis will disrupt the overlying skin [2]. Unrecognized and inadequately treated open fractures can lead to the development of osteomyelitis, premature growth arrest, and deformity [1,4,7].

Consistent with our patient, many children sustain this injury while barefoot and report bleeding from the base of the nail after stubbing their toe. Kensinger et al. reported five pediatric cases in which each patient recalled bleeding from around the nail at the time of injury [5]. In three of the patients, the presentation was delayed up to two weeks after the injury, and those patients presented with increasing pain, erythema, ascending lymphangitis, and often purulent drainage from the base of the nail [4,5]. Two of those patients had findings of osteomyelitis seen on radiographs in conjunction with the Salter-Harris I fracture of the distal phalanx of the great toe. These patients required a prolonged course of intravenous antibiotics after irrigation and debridement of the open fracture. It was noted that the remaining two patients who presented to the emergency department or pediatrician on the day of their injury underwent acute irrigation and debridement after prompt recognition of an open fracture and were successfully treated with oral antibiotics [5].

**Figure 5:**
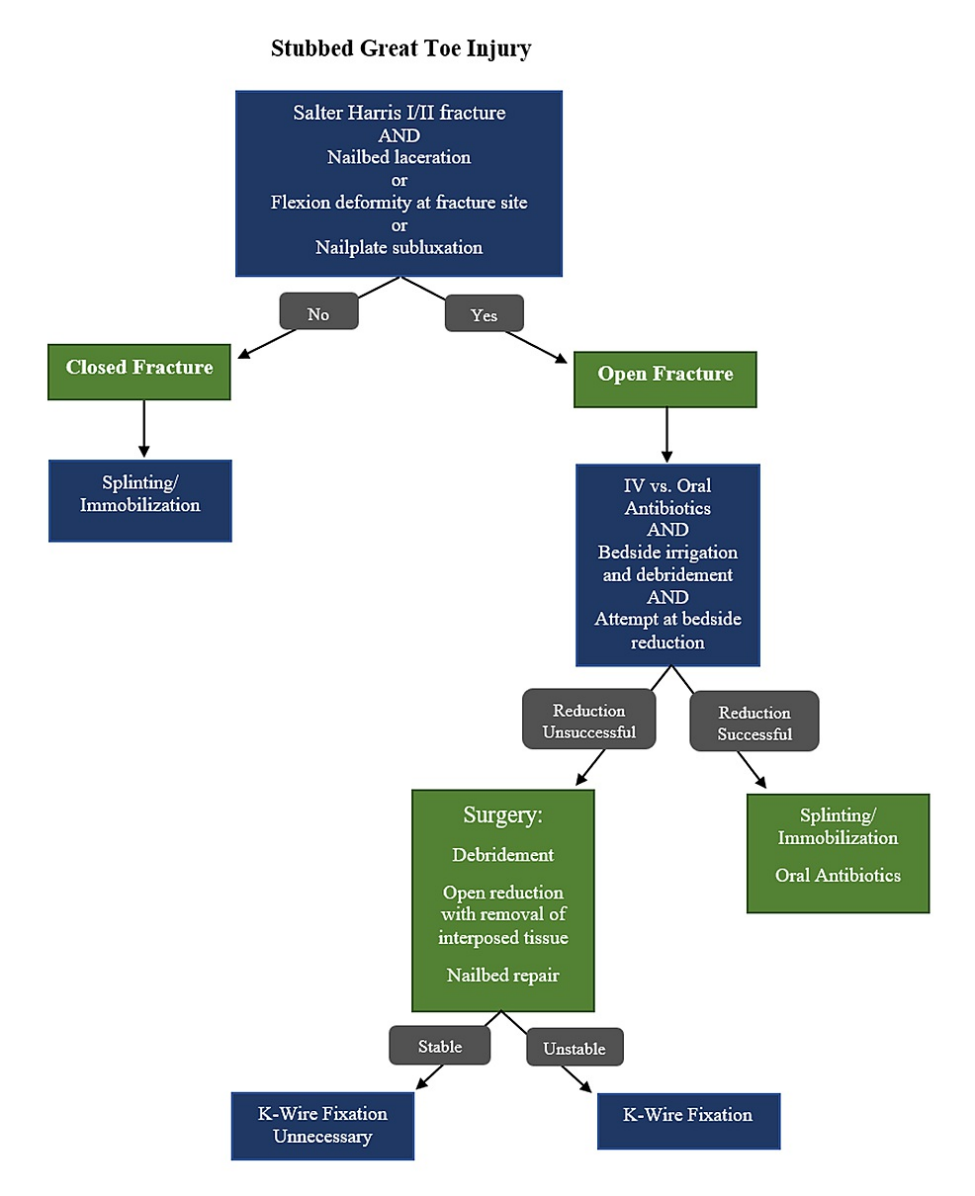
A stubbed great toe treatment algorithm.

Our patient presented for definitive management sub-acutely after having received initial treatment with oral antibiotics, irrigation with chlorhexidine scrub, and splinting in situ in an urgent care setting. After assessment of the injury in the clinic four days later, a reduction attempt was performed under a digital block, but the fracture was unable to be reduced. It was suspected that the germinal matrix was blocking the reduction, so given the nature of the open fracture, involvement of the physis, and amount of displacement, the decision was made to proceed with open reduction and pinning in the operating room. After addressing the soft tissues, the fracture was able to be successfully reduced and stabilized with a K-wire, which ultimately was accidentally removed at home three weeks post-operatively. At follow-up six weeks post-operatively, the patient was transitioning to regular activities without residual concern for infection and radiographs demonstrated maintenance of the fracture reduction. Ultimately, the patient’s fracture healed appropriately without concern for osteomyelitis or other complications as a consequence of his injury; however, the disorganized management of the patient’s open fracture from initial presentation to surgical treatment demonstrated the need for a clear treatment algorithm for open Salter-Harris fractures of the distal phalanx of the great toe (Figure 5).

There are few studies that describe open treatment for these injuries and scant documentation of indications for operative management of open and displaced Salter-Harris fractures of the distal phalanx of the great toe. Conversely, several studies have proposed algorithms for non-operative versus operative management of Seymour fractures, the hand counterpart to the stubbed great toe. Krusche-Mandl et al. analyzed the management of Seymour fractures and long-term follow-up results in a retrospective review of 24 patients treated operatively and conservatively [6]. From their analysis, an algorithm was designed which described operative management for all open fractures. This algorithm included debridement of the fracture site, open reduction, nailbed repair/nail plate fixation, and possible K-wire stabilization for open fractures. K-wire stabilization through the distal phalanx and DIPJ was recommended in cases of instability, primarily after nail subluxation. In long-term follow-up, there was one incident of growth disturbance after surgical management which was associated with a nail avulsion and tuft fracture, though the patient reported overall satisfaction [6].

While a rare injury, it is important to recognize and appropriately treat patients who present with Salter-Harris fractures involving the distal phalanx of the great toe. We propose a treatment algorithm for the assessment and management of stubbed great toe injuries, which can help guide non-operative and operative treatment decisions (Figure 5). We propose a treatment algorithm for the assessment and management of stubbed great toe injuries, which can help guide non-operative and operative treatment decisions (Figure 5). The anatomy of the distal toe predisposes the skin overlying the fracture to become easily disrupted during the injury, leading to an open fracture. Initial management should consist of prophylactic antibiotics and adequate irrigation and debridement of the fracture site. Some fracture patterns may be amenable to closed reduction with the repair of the overlying nailbed in the emergency department or clinic. However, after observation of significant interposition of the germinal matrix within the fracture site, we recommend a low threshold for operative management with the removal of the interposed tissue to ensure an adequate reduction. Additionally, stabilization with a K-wire aids in the maintenance of the reduction, especially in unstable or significantly displaced fractures. This strategy may decrease the risk of development of premature growth arrest or deformity for open fractures affecting the distal phalanx of the great toe.

## Conclusions

An assessment of the management of our patient’s open distal phalanx Salter-Harris fracture of the great toe highlights the importance of vigilance when evaluating suspicious injuries and provided the opportunity to develop a treatment algorithm to standardize efficient and effective care. We aim to decrease preventable adverse outcomes such as osteomyelitis and growth disturbance by creating a systematic method for approaching these injuries.
